# Increased Apoptosis and Proliferative Capacity are Early Events in Cyst Formation in Autosomal-Dominant, Polycystic Kidney Disease

**DOI:** 10.1100/tsw.2007.274

**Published:** 2007-11-12

**Authors:** Salwa Ibrahim

**Affiliations:** Department of Internal Medicine, Cairo University, Egypt

**Keywords:** proliferation, apoptosis, interstitial inflammation, ADPKD

## Abstract

Previous studies have highlighted epithelial proliferation and apoptosis in the cyst lining as common features in animal models of cystic disease. In this study, we sought to evaluate the timing and extent of these changes in renal tissue obtained from patients with autosomal-dominant, polycystic kidney disease (ADPKD) subjected for nephrectomy for a variety of clinical indications. Cell proliferation was assessed using an antibody to proliferating cell nuclear antigen (PCNA), and apoptosis was evaluated by the use of terminal deoxynucleotidyl transferase (TdT) digoxigenin-deoxyuridine (dUTP) nick end-labeling technique (ApopTag). The origin of cystic structures was evaluated using antibodies to epithelial membrane antigen (EMA). The lineage of interstitial mononuclear cells was assessed by anti CD 45 and CD 68 monoclonal antibodies. We found an increased rate of proliferation within the epithelium, not only of cystic, but also of noncystic, tubules that was significantly higher than the corresponding values from normal kidney (*p* ≤ 0.0001). Apoptotic index values were significantly increased within the epithelium lining noncystic and cystic structures (*p* < 0.001). In the interstitium, increased proliferation and apoptosis rates were also noted. Interstitial infiltrates were dense and consisted mainly of CD 68–positive macrophages and CD 45–positive lymphocytes. The present study demonstrated that changes in cell turnover are early events in cyst formation. The observation of mild proportionate elevation of both proliferation and apoptosis values of the epithelium lining cysts explains the lack of increase risk of renal cell carcinoma in ADPKD. The development of heavy interstitial inflammation could contribute to progressive tubulointerstitial scarring, leading to progressive renal failure.

### INTRODUCTION

In autosomal-dominant, polycystic kidney disease (ADPKD), the genetic defect results in the growth of a multitude of epithelial cysts within the renal parenchyma[1]. Despite the identification of the primary gene product involved in cystic nephron changes, the exact pathogenesis of cyst formation is not entirely clear. These include cystic dilatation of renal tubules with abnormal cell proliferation, which is a crucial key factor in the enlargement of renal cysts in ADPKD, as several experimental and human data have identified epithelial hyperplasia as a necessary element in cyst development and progressive cyst expansion[2,3,4]. The protein products of PKD genes (polycystin-1 and polycystin-2) are thought to function together as part of a multiprotein, membrane-spanning complex involved in cell-cell-matrix interactions[5]. Polycystin-1 and -2 can initiate signal transduction, leading to activation of a number of downstream effectors that regulate the cell cycle events, including cell proliferation and apoptosis[5]. Recent experimental and human studies suggested that apoptosis plays a crucial role early in cystogenesis and in progressive deterioration of renal function in ADPKD[3,6,7]. To address this issue, we investigated the timing and extent of proliferation, as well as the location and quantity of apoptosis in a cohort of end-stage ADPKD patients.

Progressive deterioration in renal function is associated with a build up of inflammatory monocytes and macrophages within renal interstitium around expanding cysts and subsequent interstitial fibrosis[8]. However, little has been shown about the role of interstitial inflammation in progressive ADPKD in humans[2,3]. Therefore, we investigated the contribution of lymphocytes and macrophages to the interstitial infiltration noted in this group. Finally, we sought to examine the source of origin of cystic structures using antibodies to epithelial membrane antigen (EMA), a distal tubular marker.

### MATERIAL AND METHODS

Human tissues were obtained from 16 adult patients with ADPKD (seven females and nine males). Details of these patients are listed in Table 1, including their gender, the age at the time of nephrectomy, and indications for nephrectomy. The tissue sections were obtained from the archival material of the Department of Pathology at the Northern General Hospital, Sheffield, UK and all were nephrectomy sections. All cases had been routinely formalin fixed and paraffin embedded. Normal tissue sections from the normal pole of ten kidneys removed because of a renal tumor served as controls and were obtained from the Department of Histopathology at the Royal Hallamshire Hospital, U.K. There was no significant difference in age and sex distribution within the ADPKD and the control groups.

**TABLE 1**
**Clinical Data of the Study ADPKD Patients**

| Case | Age/Year | Gender | Reason for Nephrectomy |
|---|---|---|---|
| 1 | 55 | M | On TX |
| 2 | 46 | M | Huge kidneys, abdominal pain |
| 3 | 47 | M | Recurrent infection, hematuria |
| 4 | 57 | F | Huge kidneys |
| 5 | 53 | M | Abdominal pain |
| 6 | 63 | M | Huge kidneys, abdominal pain |
| 7 | 56 | F | Recurrent hematuria |
| 8 | 48 | M | Abdominal pain |
| 9 | 55 | F | Infected cysts |
| 10 | 46 | M | Septicemia, infected cysts |
| 11 | 53 | F | Huge kidneys |
| 12 | 46 | F | On TX |
| 13 | 37 | M | Recurrent hematuria |
| 14 | 50 | F | Abdominal pain |
| 15 | 57 | F | Huge kidneys |
| 16 | 38 | M | Huge kidneys, abdominal pain |

### Immunohistochemistry

#### *Detection of Epithelial Proliferation by Monoclonal Antibody to PCNA*

Proliferation was detected using monoclonal antibody against proliferating cell nuclear antigen (PCNA) (clone PC10, 1:50 dilution, Dako, U.K.). For antigen retrieval, either simple heating at 95°C for 15 min[9] or a microwave pretreatment method as suggested by Shi et al. was used with slight modification[10]. After deparaffinization and endogenous peroxidase blocking, slides were placed in 10 m*M* citric acid buffer (pH 6.0) and either microwaved in a 700-W commercial microwave oven at 50% power level for 15 min or incubated in the preheated water bath that is maintained at 95°C for 15 min. The slides then were cooled for 20 min at room temperature in the buffer. After a phosphate-buffered saline (PBS) wash, the blocking serum (10% goat serum) was applied for 30 min. Slides were then incubated with the anti-PCNA antibody (1:50 dilution) overnight at 4°C. After rinsing with PBS, the sections were incubated with goat antimouse IgG-HRP (1:100 dilution, Dako, U.K.) for 60 min at room temperature. The remainder of the staining procedure was performed by the use of standardized peroxidase-antiperoxidase methodology. The reactions were developed with 3-amino-9-ethylcarbazole (ACE) substrate (Sigma, Germany).

#### *Immunostaining for Epithelial Membrane Antigen (EMA)*

Immunolocalization of epithelial cells expressing EMA, which is a distal nephron, distal convoluted tubule, and collecting duct marker was performed using monoclonal antibodies to EMA (1:300, Dako, U.K.).

#### *Immunohistochemical Staining for Interstitial Mononuclear Cells*

Immunostaining for CD 68– and CD 45–positive cells was performed using mouse antihuman CD 68 and CD 45 monoclonal antibodies diluted to 1:200 (Dako-Dakopatts, Denmark).

#### *In situ End Labeling for the Detection of Apoptotic Cells*

In histological sections, fragmented nuclear DNA associated with apoptosis were labeled *in situ* with digoxigenin-deoxyuridine (dUTP), introduced by terminal deoxynucleotidyl transferase (TdT), according to standard method described by Gavrieli et al.[11] using ApopTag® peroxidase *in situ* apoptosis detection kit (Intergen, Oxford, U.K.). Paraffin sections were dewaxed and rehydrated, treated with proteinase K (20 µg/ml) for 15 min and washed in PBS. Endogenous peroxidase was blocked with 3% $H_2O_2$ for 15 min. Slides were then covered with equilibration buffer and were incubated for 15 min at 37°C. This was followed by the incubation with the TdT for 60 min at 37°C. The reaction then was terminated using the ApopTag® stop buffer followed by anti-digoxigenin-peroxidase application and the labeled nuclei were detected with ACE substrate as the chromogen (Sigma, Germany).

### Evaluation of the Slides

For morphologic assessment, a cyst was defined as an abnormal epithelial-lined cavity with dimensions wider than 1 mm (that is, greater than five times the normal tubular diameter) and the cystically dialed tubule is any structure with dimension more than 0.4 mm. To assess the proliferative index (PI) of the epithelial cells lining the cysts, cystic tubules, and noncystic tubules, epithelial cell nuclei were counted manually in each 500–2000 nuclei and the percentage of positive nuclei for each type was calculated[2]. Only clearly definable nuclei with heavy and/or granular staining were identified as PCNA positive[2,4]. Interstitial cell PI was evaluated in 35 high-power (×40 stage objective) fields per section as the proliferating PCNA-expressing cells were found to have zonal distribution. The average number of positive PCNA per field was expressed as the interstitial PI. Glomerular PI was calculated by counting the number of PCNA-positive nuclei per glomerular tuft and the parietal epithelial cells in 20 random high-power field per section and the average number per field was considered as the glomerular PI. The apoptotic index (AI) values of the epithelium lining cystic and noncystic structures, the interstium, and the glomeruli were assessed by counting the number of TUNEL-positive nuclei in the same way as mentioned above. Only cells with observable morphological features of apoptosis were counted, i.e., shrunken cells with typically condensed nuclei with irregular cell shape and intact cell membrane. Discrete apoptotic bodies comprising a large dense nuclear fragment surrounded by a narrow cytoplasmic halo were given a single count. Similarly, clusters of small apoptotic bodies located near each other were also given a single count[12]. The number of EMA-positive, -negative, and partially stained cysts and cystic tubules also was counted in 20 fields per section, and the average number for each type was used to assess the phenotype of cystic structures. Mononuclear interstitial infiltration was assessed by counting the number of CD 45– and CD 68–positive cells in 20 randomly chosen 0.25 × 0.25 mm areas of tubulointerstitum in each section.

### Statistical Analysis

All data are expressed as mean ± SEM. Differences among the ADPKD and the normal control groups were assessed using either Student's t-test when data were normally distributed or Mann-Whitney test if skewed. Correlations within the groups for different values were evaluated by either Pearson correlation test or Spearman's rank test according to the distribution of variants. The null hypothesis was rejected at $p < 0.05$.

## RESULTS

### Morphological Assessment by Light Microscopy

Kidney tissues from ADPKD patients displayed moderate to marked interstitial fibrosis with heterogeneous interstitial infiltration with mononuclear cells. Some cysts and cystic tubules were lined with atrophic flattened epithelium (Fig. 1L), whereas others were lined with a hyperplastic epithelium composed of densely packed cells (Fig. 1K). The cysts were variable in size with occasional cystic dilatation of Bowmans' capsule. The glomeruli infrequently showed evidence of sclerosis and the blood vessels showed mild to moderate arteriosclerosis.

### EMA Staining Pattern

The pattern and the intensity of EMA staining were variable, and a nearly equal number of cysts and cystic tubules were either EMA positive or negative ($p$ = 0.840 and 0.195, respectively). Some cystic structures showed a heterogeneous staining pattern with variable intensity.

### Evaluation of Cellular Proliferation

The PI values of the epithelium lining the cysts, cystic tubules, and noncystic tubules were 18- to 39-fold higher than the respective normal control group ($p < 0.0005$). The PI was highest for the noncystic tubules

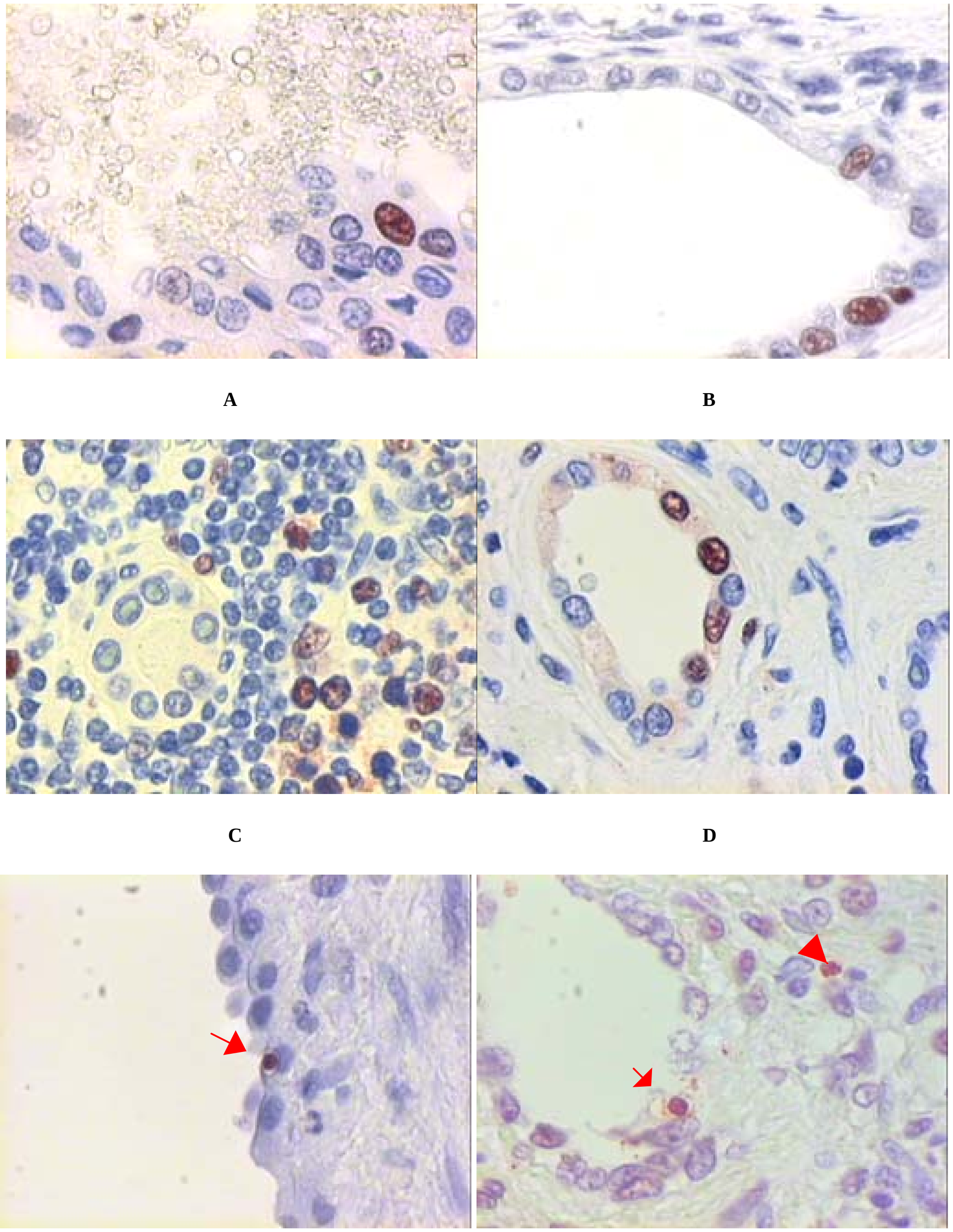

**FIGURE 1** Immunostaining of ADPKD sections. (A) PCNA-positive nucleus in the hyperplastic epithelium of a cyst. (B) Multiple PCNA-positive nuclei in the epithelium lining of a cystic tubule. (C) Multiple PCNA-positive nuclei in the epithelium lining a noncystic tubule. (D) A cluster of PCNA-positive nuclei in the interstitium around a noncystic tubule. (E) Apoptotic cell (arrow) within the hyperplastic epithelial cells lining a cyst. (F) Apoptotic cell in the epithelial lining a cystic tubule (arrow) and in the surrounding interstitium (arrowhead). (G) Apoptotic cell in the epithelial lining a noncystic tubule (arrow) and in the surrounding interstitium (arrowhead). (H) A cluster of apoptotic cells located in the interstitium (arrows). (I) Dense interstitial infiltration with CD 68–positive macrophages (using anti CD 68 antibodies). (J) Dense interstitial infiltration with CD 45–positive lymphocytes (using anti CD 45 antibodies). (K) Densely stained hyperplastic epithelium of a cyst using antibodies to EMA. (L) A cyst lined with atrophic epithelium showed negative EMA staining (magnification ×400).

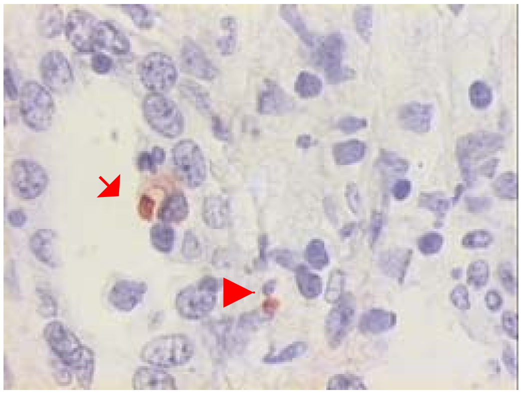

FIGURE 1G

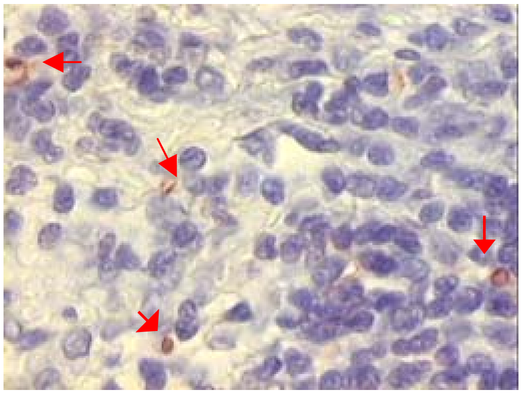

FIGURE 1H

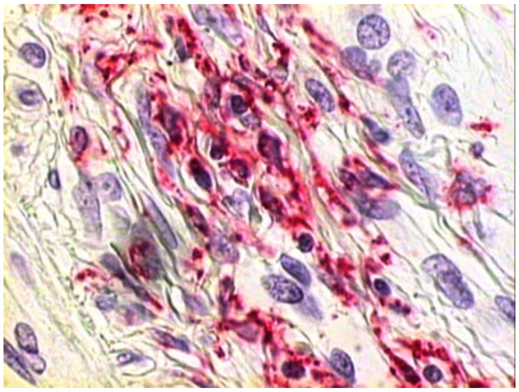

FIGURE 1I

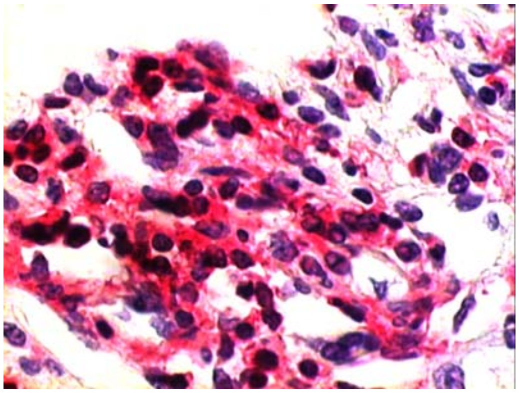

FIGURE 1J

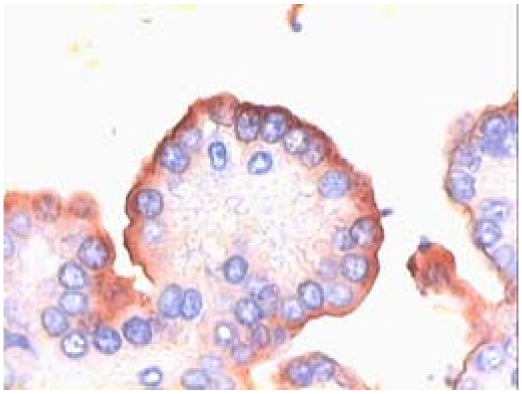

FIGURE 1K

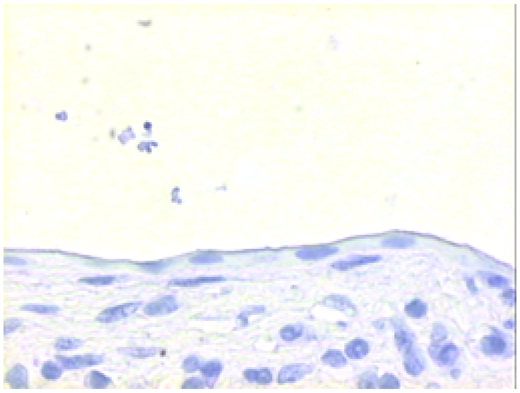

FIGURE 1L

that was 39-fold higher than the corresponding normal control (1.19 ± 0.15 and 0.03 ± 0.02, respectively) (Fig. 2). Of note, the PI value of the epithelial lining noncystic tubules was also higher than that lining the cysts (1.19 ± 0.15 and 0.47 ± 0.09, $p < 0.001$). The PI values showed no consistent correlation with the epithelial morphology, as it was noted that the hyperplastic epithelium did not show a higher degree of proliferation compared to other types. There was a great variation in the number of PCNA-positive nuclei between individual cystic and noncystic structures in the same and between the sections, and, not infrequently, clusters of PCNA-positive nuclei were noted grouped in the cystic and noncystic structures as well as the interstitium nearby.

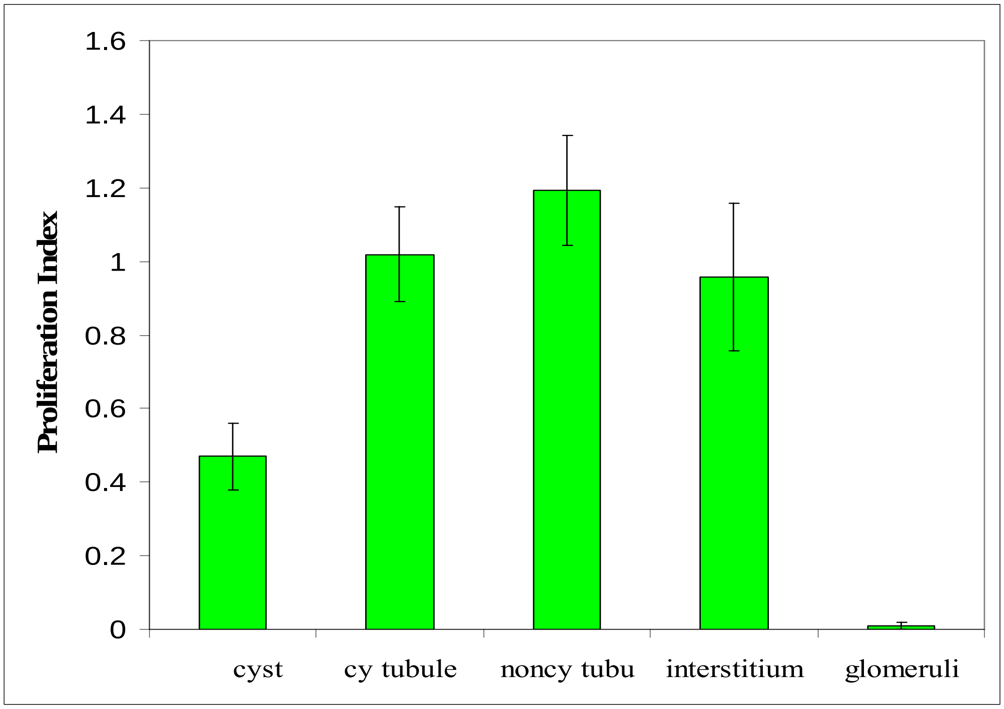

**FIGURE 2** The PI in ADPKD tissues (mean ± SEM).

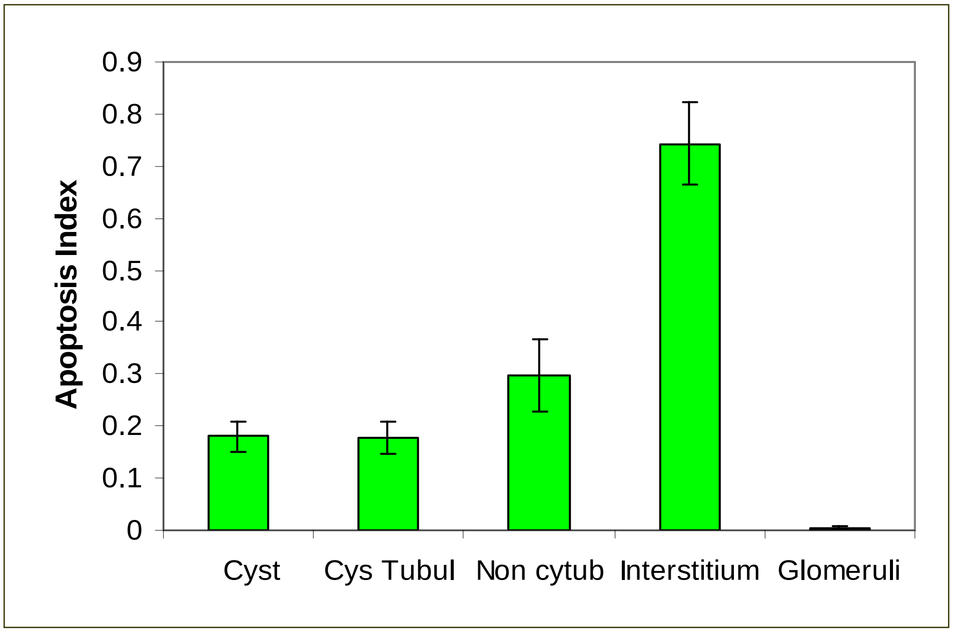

**FIGURE 3** The AI of ADPKD sections (mean ± SEM).

Of note, the interstitium displayed a similar high degree of proliferation that was significantly higher than the corresponding value of normal control (0.95 ± 0.20 and 0.005 ± 0.005, respectively, $p < 0.0005$). The PI was higher in the interstitium adjacent to the cysts and cystic tubules where clusters of 2–10 PCNA-positive nuclei were encountered more frequently (Fig. 1).

### Evaluation of Apoptosis in ADPKD

The AI values of the epithelial lining of cysts, cystic tubules, noncystic tubules, and the interstitium were markedly increased compared with the respective control group ($p \leq 0.001$)(Fig. 3). The interstitial apoptotic score was significantly higher than that of the epithelium lining the noncystic and cystic structures ($p < 0.0001$) and was about 29.7-fold increased over controls. Apoptotic cells were found either as singles cells or clusters of cells in the cystic epithelium or in the lumen of the cyst (Fig. 1E). In the interstitium, apoptotic cells were found to occur either as single cells scattered sparsely or as clusters adjacent to the surrounding cysts and cystic tubules (Fig. 1H). There was no statistically significant difference between the apoptotic score of the glomeruli in ADPKD group and the normal control.

### Correlation between Cell Proliferation and Apoptosis

ADPKD tissues displayed coupled stimulation of apoptosis and proliferation with predominance of the later. No significant correlation, however, was found between the proliferation and the apoptosis scores in each renal compartment ($p > 0.05$).

### Interstitial Infiltrates in ADPKD Patients

Interstitial infiltrates were dense and heterogeneous in distribution. Immunostaining revealed a predominance of CD 68–positive macrophages (27.9 ± 5.6 cells/mm$^2$, $p < 0.005$) and CD 45–positive lymphocytes (17.3 ± 2.1 cells/mm$^2$, $p < 0.005$).

## DISCUSSION

A large body of experimental and human studies identified epithelial hyperplasia as a necessary element in the process of cyst formation and expansion in PKD[13,14,15,16]. In this study, the PI value as determined by the number of PCNA-positive nuclei was substantially higher in the epithelium lining the noncystic tubules and was 39-fold higher than the corresponding control value ($p < 0.0005$). Of interest, the PI value of the epithelial cells lining noncystic tubules was higher than that of the cyst ($p < 0.001$). This was described first by Nadasdy et al. as a field effect in the nephron in the sense that a certain segment of the nephron seems primarily to be involved in increased epithelial proliferation that appears to precede cyst formation[2].

The PI of the epithelium lining the cystic tubules was 30-fold higher than the corresponding control value ($p < 0.0005$), while that of cyst was about 18-fold higher than the corresponding control value ($p$=0.0001). No consistent correlation with a specific epithelial morphology was detected, as cysts with hyperplastic-layered epithelium did not display higher PI than that of those with attenuated atrophic cell lining. These results are consistent with previous immunohistochemical studies[2,3] and may also explain the lack of consistent increase in the risk for renal cell carcinoma in ADPKD despite the high epithelial proliferative rate.

One of the key findings in this study was the demonstration of a significant increased interstitial PI that was about 190-fold higher than the corresponding control value ($p < 0.0005$). The PCNA-positive nuclei were noted either in clusters located adjacent to the expanding cysts and cystic structures or sparsely in the interstitium. In addition, the interstitial infiltrates were dense and consisted mainly of CD 68–positive macrophages and CD 45–positive lymphocytes ($p < 0.005$). This interstitial inflammation reflects one of the basic fundamental features that is common to all species of PKD[8,18]. Progressive deterioration in renal function is associated with a buildup of inflammatory monocytes and macrophages within the renal interstitium around the expanding cysts[8]. The monocyte/macrophage influx plays a major role in the induction of myofibroblasts with subsequent interstitial fibrosis[18]. There is also increasing evidence to suggest that cystic fluid components could potentiate monocyte chemotaxis and progressive interstitial inflammation and fibrosis in human PKD[19,20]. It is noteworthy that three of the examined kidneys were removed because of recurrent infection or infected cysts/septicemia (case 3,9,10). The existence of cyst infection could, of course, intensify the extent of tubulointerstial inflammation detected in this study and may explain why these cases have progressed to end-stage kidney disease. However, we found heavy pericystic infiltration by macrophages and lymphocytes in other kidney tissues examined, thus highlighting the impact of expanding cysts and their chemokines content, whether infected or not, on the neighboring interstitium.

There was marked variability in the number of PCNA-positive nuclei within and between the sections, even in the controls. This was reported previously and may result from local differences in the speed of epithelial cell turnover and/or the variability of the growth rate of the individual cyst[2]. In addition, the variable rate of acquisition of somatic changes inactivating the normal PKD1 and PKD2 alleles could further explain the focal nature of the proliferation and cyst transformation in ADPKD[17].

Transgenic and targeted mutagenesis approaches have given direct proof that cystogenesis hinges on the critical imbalance between cell proliferation and apoptosis[21,22,23]. It was thought that epithelial cells deprived of interaction with underlying matrix would develop apoptosis within hours[24]. In addition, apoptosis was suggested to play a crucial role in progressive loss of renal parenchyma in ADPKD[25,26,27]. In this study, the apoptotic index was significantly increased in the epithelium lining noncystic tubules and was 29-fold higher than the corresponding control value ($p < 0.0005$). A high AI was also significantly increased in the epithelium lining cystic structures, although to a lesser degree ($p \leq 0.001$). Similarly, the interstitial apoptotic score was significantly higher than that of the control (29.7-fold, $p < 0.0005$), and was located either as single cells or in clusters adjacent to the noncystic and cystic structures.

In this study, epithelial and interstitial cellular proliferation was coupled with increased apoptosis with the predominance of the former. However, there was no significant correlation between the degree of apoptosis and proliferation in each renal compartment ($p > 0.05$). These data confirm the observation reported before that proliferation and fluid secretion in polycystic kidney epithelia outweigh the high levels of cell death reported, hence causing increased size of the kidneys with concomitant destruction of renal tissue[7]. This may also reflect the discrepancy between the half-life of apoptotic and proliferating cells. Apoptosis is a short-term event because within solid tissues, apoptotic cells are phagocytized within few hours by neighboring cells[28]. On the other hand, the time necessary for a complete proliferative cell cycle is much longer, with the detection half-life of PCNA protein being ~20 h[29,30]. Therefore, it would be more accurate if we had assessed the mitotic morphology or looked at a cell division marker like cyclin D2 simultaneously. Similarly, morphologic assessment for apoptosis by light microscopy could exclude the possibility of detection of random nuclear fragmentation by TUNEL technique late in the course of cellular necrosis[31].

Finally, we sought to determine the source of origin of the cystic structures using EMA, which is a distal tubular marker. An equal proportion of cysts and cystic tubules were either EMA positive or negative with some cysts showing a heterogeneous staining pattern with variable intensity. These findings are consistent with previous observations that ADPKD cysts may originate from any nephron segment[32]. However, we did not assess the cysts directly for specific proximal tubular markers. Thus, EMA-negative cysts could either originate from proximal tubules or represent dedifferentiated distal tubular cells.

To conclude, the present study has shown that changes in cell turnover are early events in cyst formation and may contribute to cyst expansion. The presence of heavy interstitial inflammation in the ADPKD tissues could explain the development of tubulointerstitial scarring, leading to progressive renal failure in some PKD patients.
